# Comparison of Digestive Enzyme Activities and Expression of Small Intestinal Transporter Genes in Jinhua and Landrace Pigs

**DOI:** 10.3389/fphys.2021.669238

**Published:** 2021-06-14

**Authors:** Xiuting Liu, Wentao Lyu, Lei Liu, Kaikai Lv, Fen Zheng, Yuanxia Wang, Jinggang Chen, Bing Dai, Hua Yang, Yingping Xiao

**Affiliations:** ^1^State Key Laboratory for Managing Biotic and Chemical Threats to the Quality and Safety of Agro-Products, Institute of Agro-Product Safety and Nutrition, Zhejiang Academy of Agricultural Sciences, Hangzhou, China; ^2^Zhejiang Goshine Test Technologies Co., Ltd., Hangzhou, China; ^3^Agricultural and Rural Bureau of Kaihua County, Kaihua, China

**Keywords:** Jinhua pigs, Landrace pigs, digestive enzyme activities, transporter gene, mRNA levels

## Abstract

Digestive enzyme activity is involved in the regulation of growth performance because digestive enzymes function to improve the feed efficiency by digestion and in turn to modulate the process of nutrient metabolism. The objective of this study was to investigate the differences of the digestive enzyme activities and expression of nutrient transporters in the intestinal tract between Jinhua and Landrace pigs and to explore the potential breed-specificity in digestion and absorption. The pancreas segments and the digesta and mucosa of the duodenum, jejunum, and ileum were collected from 10 Jinhua pigs and Landrace pigs, respectively. The activities of trypsin, chymotrypsin, amylase, maltase, sucrase, and lipase were measured and the expression levels of *PepT1*, *GLUT2*, *SGLT1*, *FABP1*, *FABP2*, and *FABP4* were examined. Results showed that the trypsin activity in the pancreas of Jinhua pigs was higher than that in Landrace pigs, but was lower in the small intestine, except for in the jejunal mucosa. The chymotrypsin activity in the small intestine of Jinhua pigs was higher than that in Landrace pigs, except for in jejunal mucosa and contents. Compared with Landrace pigs, the amylase and maltase activity in the small intestine of Jinhua pigs was lower, except for in ileal mucosa. The sucrase activity in the small intestine of Jinhua pigs was also lower than Landrace pigs, except for in jejunal mucosa. Furthermore, the lipase activity in the small intestine of Jinhua pigs was higher than that in Landrace pigs. The mRNA levels of *PepT1* and *GLUT2* in duodenal, jejunal and ileal mucosa showed no difference between Jinhua and Landrace pigs, whereas *SGLT1* in ileal mucosa was lower in Jinhua pigs. The mRNA levels of *FABP1*, *FABP2* and *FABP4* in the small intestinal mucosa of Jinhua pigs were higher than in Landrace pigs. These findings indicate that there is a certain difference in the digestibility and absorption of nutrients in small intestine of Jinhua and Landrace pigs, partially resulting in their differences in growth development and fat deposition.

## Introduction

Pigs is one of the most essential livestock species and serves as a major food source as well as an ideal animal model to study human diseases due to the high similarity with humans in the anatomical structure, physiology, biochemical index, food structure, and drug metabolism (Miller and Ullrey, 1987; Baker, 2008; Xiao et al., 2018). Nutrient digestion in animals includes physical digestion, chemical digestion and microbial fermentation. Chemical digestion involving digestive enzymes is the main link in the whole digestion process (Yen et al., 1991). The digestive enzyme activity affects the digestive efficiency of nutrients, which in turn modulates the process of nutrient metabolism. Therefore, digestive enzyme activities play an important role in the growth performance. Barea et al. (2011) found that different genotypes had different overall energy use efficiencies and different energy and protein deposition in tissues due to the differences in the small intestinal structures and nutrient digestibility. Urriola and Stein (2012) studied the difference in the digestibility of fiber diets for different breeds of pigs (Meishan pigs and Yorkshire pigs). They found that the apparent digestibility of dry matter, the nutrients in soybean meal diets and dry distillers’ grains and their soluble diets in Meishan pigs were higher than Yorkshire pigs. Therefore, different pig breed gives different digestion rate and nutrient utilization. However, whether this difference is related to digestive enzyme activities remains unclear (Beck, 1973; Lindemann et al., 1986).

After the digestion, nutrients, such as protein, sugar and fat, are absorbed through transporter proteins. Oligopeptides (dipeptides/tripeptides) are the main form of the protein digestion products, which can be transported and absorbed by the oligopeptide transporter (*PepT1*). Sodium-glucose cotransporter 1 (*SGLT1*) and glucose transporter 2 (*GLUT2*) play an important role in sugar absorption in the small intestine (Wright et al., 1991). All members of the fatty acid-binding proteins (*FABPs*) family have the most basic function of managing fatty acid absorption and intracellular transport and regulating fat metabolism in animals.

The Jinhua pig, one of the most important Chinese indigenous pig breeds, exhibits an earlier sexual maturity, lower growth rate, higher fat content and lower lean meat content than western pig breeds such as Landrace pigs (Miao et al., 2009; Wu et al., 2013; Huang et al., 2019). To date, most studies related to pigs have focused on porcine growth performance and meat quality, whereas the underlying digestive mechanism has not been fully studied and few comprehensive studies on metabolites have been published (Gan et al., 2020; Martins et al., 2020; Wang et al., 2021). Our study compared and analyzed the activities of digestive enzymes and the mRNA expression levels of related transporter genes in the pancreas and small intestine of Jinhua and Landrace pigs under the same feeding conditions and explored the possible relationship of the digestive enzyme activity and transporter genes in Jinhua and Landrace pigs, which might lead to their different characteristics.

## Materials and Methods

### Animals and Sample Collection

All animal procedures were approved by the Institutional Animal Care and Use Committee of the Zhejiang Academy of Agricultural Sciences, and all methods were performed in accordance with the relevant guidelines and regulations.

Our test animals were the same as described in our previous report (Xiao et al., 2018). Briefly, 36 weanling piglets, including Jinhua and Landrace pigs, were housed in six pens in an environmentally controlled room and fed a commercial diet *ad libitum* under standard management, with six pigs housed in a single pen. Five healthy male and five female pigs of similar weight were selected from each breed and slaughtered on day 240. The digesta and mucosa from the middle of the pancreas, duodenum, jejunum, cecum, and ileum were collected (except for the duodenal digesta), snap frozen in liquid nitrogen, and stored at −80°C.

### Digestive Enzyme Activity Analysis

The pancreatic tissue, small intestinal mucosa and digesta samples were thawed and homogenized in 0.9% ice-cold NaCl solution which was nine times larger in volume than the determining samples. Then the homogenates were centrifuged at 4°C for 10 min at 2,500 rpm to obtain the supernatants. The activities of trypsin, chymotrypsin, amylase, maltase, sucrase, and lipase were measured by commercial assay kits (Nanjing Jiancheng Bio-Engineering Institute, Nanjing, China) according to the instructions of manufacturer (Deng et al., 2020). In addition, the protein concentration was assessed using commercial kits (Nanjing Jiancheng Bioengineering Institute, Nanjing, China) following the manufacturer’s guidelines. Enzyme activities were expressed in units per milligram or gram of protein.

### RNA Extraction

RNA was extracted and purified from each intestinal mucosa using the RNase-Free DNase Set (Qiagen, Germany) and the TRIzol^®^ Plus RNA Purification Kit (Thermo Fisher Scientific, United States) according to the manufacturer’s instructions. Then, the extracted RNA was reverse transcribed into cDNA using the SuperScript^TM^ III First-Strand Synthesis SuperMix for qRT-PCR kit (Thermo Fisher Scientific, United States) according to the manufacturer’s instructions.

### Real-Time Quantitative PCR (RT-PCR)

The mRNA expression levels of *PepT1*, *GLUT2*, *SGLT1*, *FABP1*, *FABP2*, and *FABP4* in the small intestinal mucosa were measured by the Power SYBR^®^ Green PCR Master Mix (Thermo Fisher Scientific, United States) and a CFX384 multiplex real-time fluorescence quantitative PCR system (Bio-Rad, United States). The reaction conditions were as follows: predenaturation at 95°C for 1 min, denaturation at 95°C for 15 s, annealing at 63°C for 25 s and fluorescence collection. The primers were designed by Primer Premier 6.0 software, and synthesized by Bioengineering Biotechnology (Shanghai) Co., Ltd. Primer information for genes chosen for confirmation of expression using RT-PCR are shown in Table 1. In the present study, glyceraldehyde-3-phosphate dehydrogenase (GAPDH) gene was selected as a reference, whose expression level did not differ in different tissues. The relative expression levels were normalized to the GAPDH gene and expressed as fold change (Wang et al., 2021). The 2^–Δ^ ^Δ^ ^*Ct*^ method was used to calculate relative expression levels (Livak and Schmittgen, 2001).

**TABLE 1:** Primer information for genes chosen for confirmation of expression using RT-PCR.

Gene name	Gene library sequence number	Primer sequence (5′ to 3′) (F: former primer, R:reverse primer)	Size (bp)
*PepT1*	NM_214347.1	F:GCAGACCGTCAACGCCATCCT	125
		R:GGAACATCCCAACTGTCATCTTCCT	
*SGLT1*	NM_001164021.1	F:CCCAGCAACTGTCCCACAATT	135
		R:GCGGTAGAGATGCACATCTGGAA	
*GLUT2*	NM_001097417.1	F:CGGTGGGACTTGTGCTACTGGA	146
		R:GCGTGGTCCTTGACTGAAAAACT	
*FABP1*	NM_001004046.2	F:TGAACTCAACGGTGACATA	75
		R:ATTCTCTTGCTGATTCTCTTG	
*FABP2*	NM_001031780.1	F:CTCGCAGACGGAACTGAACTCA	127
		R:CCATTTCATCCCCGATAATTTCT	
*FABP4*	NM_001002817.1	F:TGGAAACTTGTCTCCAGTG	147
		R:GGTACTTTCTGATCTAATGGTG	
*GAPDH*	AF017079	F:GGCAAATTCCACGGCACAGTCA	82
		R:CTCGCTCCTGGAAGATGGTGAT	

### Statistical Analyses

The experiment data were analyzed using unpaired Students’ two-tailed t-test in SPSS 20.0. *P* < 0.05 was considered significant while *P* < 0.01 was considered extremely significant when. The charts were drawn with GraphPad Prism version 7.0. Results are expressed as mean ± SEM.

## Results

### Comparison of Protease Activities in Jinhua and Landrace Pigs

**FIGURE 1:**
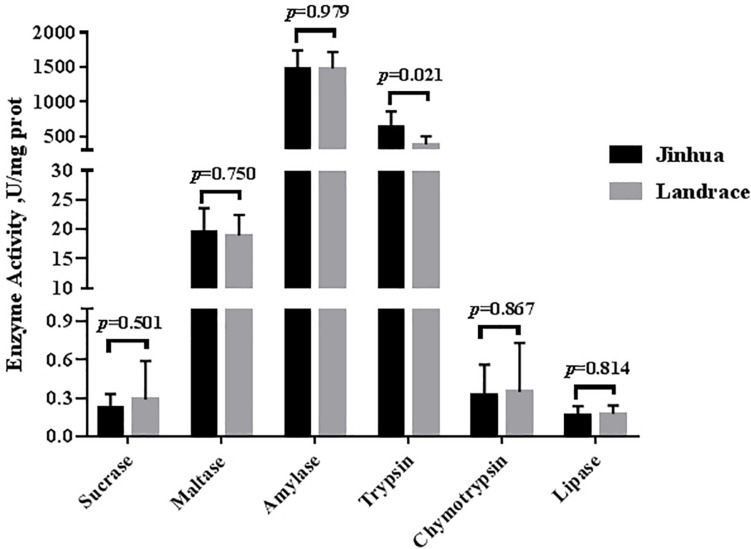
Comparison of the activities of trypsin, chymotrypsin, amylase, maltase, sucrase, and lipase in the pancreas of Jinhua and Landrace pigs. The pancreas was collected from 10 Jinhua pigs and 10 Landrace pigs for the determination of the trypsin, chymotrypsin, amylase, maltase, sucrase, and lipase activities. Data were expressed as mean ± SEM. The statistics was performed using unpaired Students’ two-tailed *t*-test.

**FIGURE 2:**
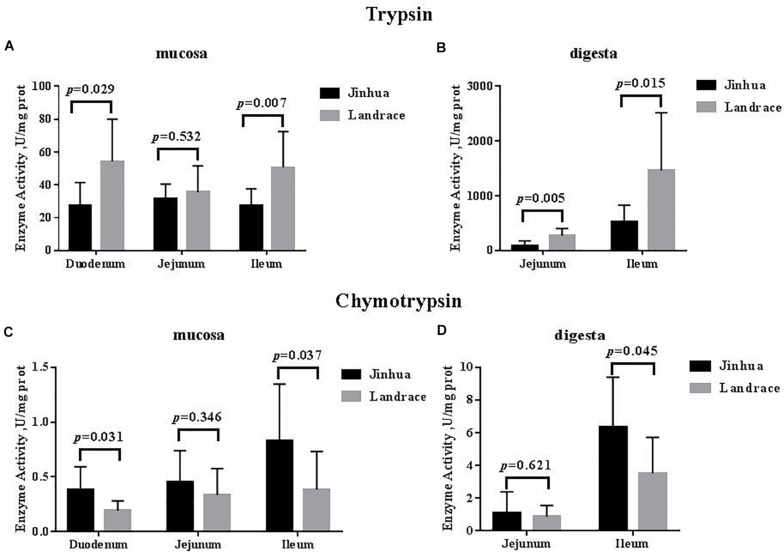
Comparison of the activities of trypsin and chymotrypsin in the small intestine of Jinhua and Landrace pigs. The mucosa and digesta of duodenum, Jejunum, and ileum were collected from 10 Jinhua pigs and 10 Landrace pigs for the determination of the trypsin **(A,B)** and chymotrypsin **(C,D)** activities. Data were expressed as mean ± SEM. The statistics was performed using unpaired Students’ two-tailed *t*-test.

As shown in Figures 1, 2, the trypsin activity in the pancreas of Jinhua pigs was higher than that in Landrace pigs (*P* < 0.05) but was lower in the duodenal mucosa, jejunal content, ileal mucosa, and ileal content of Jinhua pigs (*P* < 0.05), with no significant difference in the jejunal mucosa (*P* > 0.05). The chymotrypsin activity in the duodenal mucosa, ileum mucosa and content of Jinhua pigs was higher than those in Landrace pigs (*P* < 0.05). However, there was no difference in the chymotrypsin activity in the pancreas, jejunal mucosa and contents between Jinhua and Landrace pigs (*P* > 0.05).

### Comparison of the Activities of Carbohydrate Digestive Enzymes in Jinhua and Landrace Pigs

**FIGURE 3:**
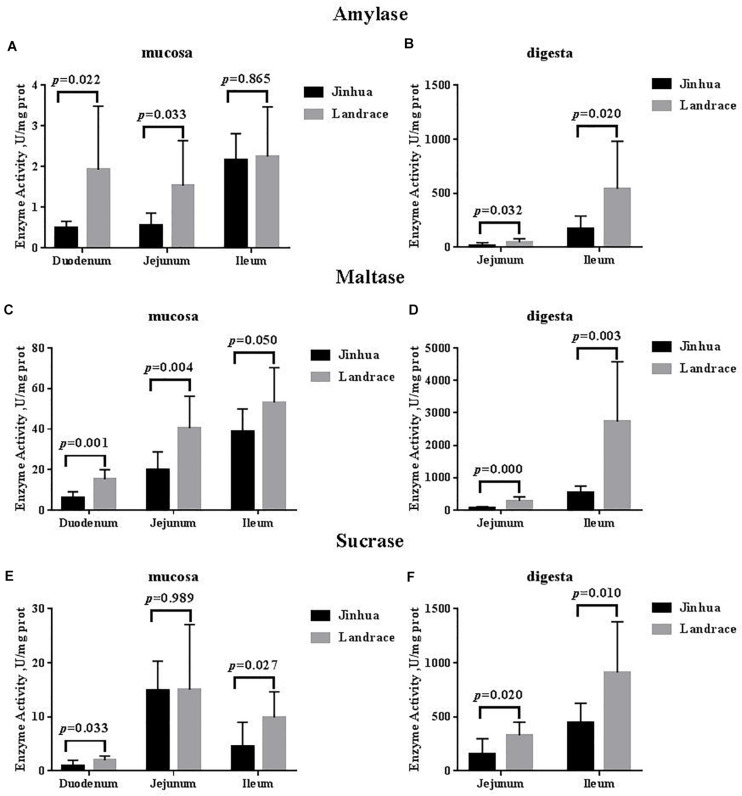
Comparison of the activities of amylase, maltase, and sucrase in the small intestine of Jinhua and Landrace pigs. The mucosa and digesta of duodenum, Jejunum, and ileum were collected from 10 Jinhua pigs and 10 Landrace pigs for the determination of the amylase **(A,B)**, maltase **(C,D)**, and sucrase **(E,F)** activities. Data were expressed as mean ± SEM. The statistics was performed using unpaired Students’ two-tailed *t*-test.

There were no differences in the amylase, sucrase and maltase activities in the pancreas of Jinhua and Landrace pigs (*P* > 0.05; Figures 1, 3). In the duodenal mucosa, the amylase, maltase and sucrase activities of Jinhua pigs were lower than those of Landrace pigs (*P* < 0.05). In the jejunal mucosa, the amylase and maltase activities of Jinhua pigs was lower than Landrace pigs (*P* < 0.05) while the sucrase activity showed no difference (*P* > 0.05). In the jejunal content, the amylase, sucrase and maltase activities of Jinhua pigs were lower than Landrace pigs (*P* < 0.05). In the ileal mucosa, the maltase and sucrase activities of Jinhua pigs were lower than Landrace pigs (*P* < 0.05), whereas the amylase activity showed no difference (*P* > 0.05). In the ileal contents, the activities of amylase, sucrase and maltase in Jinhua pigs were lower than Landrace pigs (*P* < 0.05).

### Comparison of Lipase Activity in Jinhua and Landrace Pigs

**FIGURE 4:**
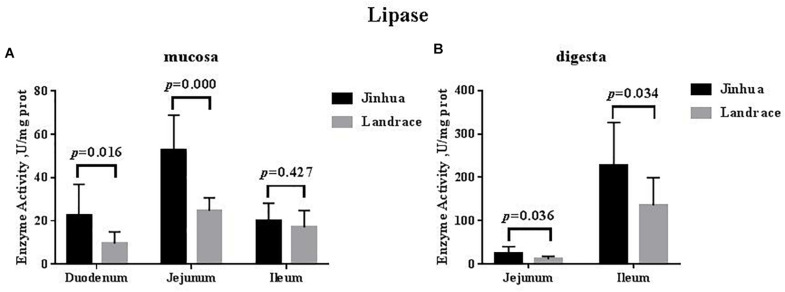
Comparison of the lipase activity in the small intestine of Jinhua and Landrace pigs. The mucosa **(A)** and digesta **(B)** of duodenum, Jejunum, and ileum were collected from 10 Jinhua pigs and 10 Landrace pigs for the determination of the lipase activity. Data were expressed as mean ± SEM. The statistics was performed using unpaired Students’ two-tailed *t*-test.

The lipase activity in the duodenal mucosa, jejunal mucosa, jejunal content, and ileal content of the Jinhua pigs was higher than those in Landrace pigs (*P* < 0.05), whereas no difference in the lipase activity was observed in the pancreas of Jinhua and Landrace pigs (Figures 1, 4).

### Comparison of the Expression Levels of *PepT1*, *GLUT2*, *SGLT1*, FABP1, *FABP2*, and *FABP4* in the Intestinal Mucosa of Jinhua and Landrace Pigs

**FIGURE 5:**
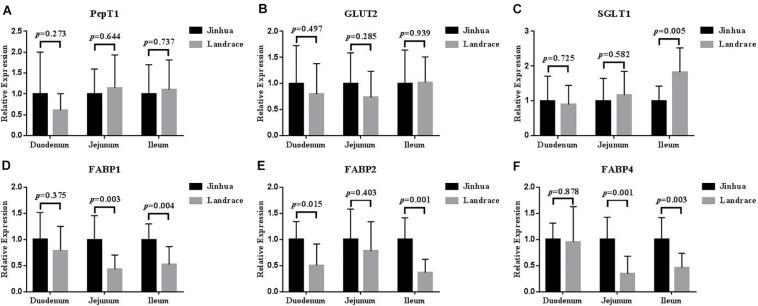
Relative expression levels of *PepT1*, *GLUT2*, *SGLT1*, *FABP1*, *FABP2*, and *FABP4* in the duodenum, jejunum, and ileum. The segments of duodenum, Jejunum, and ileum were collected from 10 Jinhua pigs and 10 Landrace pigs to examine the expression levels of *PepT1* **(A)**, *GLUT2* **(B)**, *SGLT1* **(C)**, *FABP1* **(D)**, *FABP2* **(E)**, and *FABP4* **(F)** by using RT-PCR analysis. GAPDH was used as the reference gene. Data were expressed as mean ± SEM. The statistics was performed using unpaired Students’ two-tailed *t*-test.

The mRNA expression levels of *PepT1* and *GLUT2* in the duodenal, jejunal and ileal mucosa showed no difference between Jinhua and Landrace pigs (*P* > 0.05; Figure 5), whereas the expression of *SGLT1* in the ileal mucosa was lower in Jinhua pigs than in Landrace pigs (*P* < 0.01; Figure 5). The mRNA levels of *FABP1*, *FABP2*, and *FABP4* in all of the small intestinal mucosa of Jinhua pigs were higher than those in Landrace. In detail, *FABP1* expression in the jejunal and ileal mucosa of Jinhua and Landrace pigs showed an extremely significant difference (*P* < 0.01). The expression of *FABP2* showed a significant difference in the duodenal mucosa (*P* < 0.05) and an extremely significant difference in the ileal mucosa (*P* < 0.01) between Jinhua and Landrace pigs. *FABP4* expressed in the jejunal and ileal mucosa of Jinhua and Landrace pigs gave an extremely significant difference (*P* < 0.01).

## Discussion

Various digestive enzymes secreted by the small intestine and pancreas play a catalytic and regulatory role in the process of digesting various nutrients in the diet. Digestive enzymes mainly include protease, amylase, and lipase. Trypsin and chymotrypsin are the most important protein digestive enzymes in the animal intestine. Their activities are important indexes to reflect the protein digestive capacity in animals (Kluge et al., 2006). Amylase is involved in carbohydrate catabolism and affects the digestion and absorption of carbohydrates (Courtois et al., 2002). The digestive enzyme activity in the gastrointestinal tract is closely related to the digestive ability of the different nutrients and production performance in pigs (Lindemann et al., 1986; Jensen et al., 1997; Urriola and Stein, 2012; Deng et al., 2020). The results of this study found that most digestive enzyme activities in the pancreatic tissue of Jinhua and Landrace pigs showed no significant difference, except for the trypsin. Previous studies showed that the digestive enzyme activities in the pancreas of different pig breeds in the early stage were very low and that there were no significant differences among the breeds (Owsley, 1982; Lindemann et al., 1986). Lindemann et al. (1986) found the trypsin activity in the pancreas was significantly reduced in piglets at 30 days old. Nevertheless, studies by Jensen et al. (1997) and Makkink et al. (1994) found pancreatic digestive enzyme activities did not differ before and after weaning in long × large binary pigs. Therefore, the digestive enzyme activities in the pancreas may have differences among species and developmental stages, which might result from dietary nutrition levels, feeding management methods, age, sampling, and other factors.

The small intestine plays a key role in animal digestion and absorption of dietary nutrients (King et al., 2000; Adeola and King, 2006). Most nutrients reach the small intestine and are eventually broken down into small molecules that can be directly absorbed by the body (Smoot and Findlay, 2000). Trypsin and chymotrypsin belong to the serine protease family and exist in the digestive system. Trypsin decomposes proteins into peptides by specifically identifying peptide bonds formed by arginine and lysine carboxyl segments. Chymotrypsin mainly decomposes the protein or polypeptide into small peptides and amino acids (Kemp et al., 1991; Deng et al., 2020). Chymotrypsin acts mostly on the peptide bond composed of the carboxyl group of the aromatic amino acid, with a small amount of activity on the peptide bond formed by leucine, glutamine, and methionine. Jensen et al. (1997) found that the chymotrypsin activity decreased in the small intestine after weaning without changes in trypsin activity. The earlier the weaning time, the longer the time required for chymotrypsin activity to return to normal levels in the gastrointestinal tract of weaned piglets, suggesting that chymotrypsin activity may be affected by diet and stress (Lindemann et al., 1986). In our study, the trypsin activity in the small intestine of Jinhua pigs was significantly lower than that in Landrace pigs except for the jejunal mucosa, which indicates that the Jinhua pig, as a local fat-type pig (Xiao et al., 2018; Wu et al., 2021), may have a weaker ability to digest protein than the exotic lean-type pig such as the Landrace. The chymotrypsin activity of Jinhua pigs was higher than that in Landrace pigs, indicating that there was a difference in chymotrypsin activity among different species, which may be related to the degree of protein decomposition.

The key step in the sugar digestion may be the decomposition on the brush border of the intestinal epithelium (Uni et al., 1998). The starch can be only absorbed through the intestinal wall after being decomposed into monosaccharides (Riby and Kretchmer, 1985; Semenza, 1986). Therefore, disaccharidase plays an important role in the decomposition and absorption of carbohydrates. The disaccharidase on the intestinal brush border of mammals functions to digest 80% of the maltose in a combined manner from the diet (Swallow et al., 2001; Nichols et al., 2003), and its activity was related to carbohydrate intake (Shinohara et al., 1986; Samulitis-dos Santos et al., 1992). The amylase activity determines the concentration of the disaccharidase reaction substrate in the intestine, with higher activity resulting in a higher the substrate concentration. We found that the amylase and maltase activities in the small intestine except for the ileal mucosa in Jinhua pigs were lower than those in Landrace pigs, and the sucrase activity in the small intestine except for the jejunal mucosa was also lower. The Jinhua pig, may have a lower digestion capacity for carbohydrates than lean-type pigs such as the Landrace.

Digestion and absorption of fat is mainly carried out in the small intestine and is associated with lipase activity (Ville et al., 2002). The fat utilization efficiency in different breeds of piglets mainly depends on the lipase activity from the small intestine (Cera et al., 1990; Jensen et al., 1997), and after weaning, the Meishan pig’s stronger ability to utilize fat in the diet is due to the stronger lipase activity in the pancreas (Kemp et al., 1991). Our study found that the lipase activity in small intestine was higher than that in Landrace pigs, suggesting that Jinhua pigs might have a higher fat digestion capacity than Landrace pigs. The results were positively related to our previous study which found that the average backfat thickness and intramuscular fat in the longissimus muscle of Jinhua pigs was much higher than that of Landrace pigs (2.7 cm vs. 1.7 cm, *P* < 0.01; 3.74% vs. 2.55%, *P* < 0.01) (Xiao et al., 2018).

Oligopeptide transporter 1 is predominantly expressed in the intestinal epithelium and functions in the absorption of dietary nutrients (Fei et al., 1994; Liang et al., 1995). The small (oligo) peptide transport system of *PepT1* has a higher efficiency, faster absorption and other absorption advantages compared with the amino acid transport system (Vincenzini et al., 1989) so that the lack of amino acids cannot influence the mRNA levels of small peptide transporters. The regulation of *PepT1* in the small intestine is affected by the nutritional status of the body, endocrine hormones, and intestinal microecology (Adibi, 2003). Our study showed there was no difference in the expression level of *PepT1* between Jinhua pigs and Landrace pigs.

The starchy polysaccharides in food are mainly absorbed in the form of monosaccharides by the intestinal mucosa after decomposition by digestive enzymes, of which 80% are glucose (Tavakkolizadeh et al., 2005). The monosaccharide binds to *SGLT1* in the brush border of the intestinal mucosa and is transported into the cell. After accumulating to a certain concentration in the cell, the monosaccharide binds to *GLUT2* in the cell membrane in a cis concentration gradient and facilitates diffusion through the tissue basement membrane into the intercellular space (Wright et al., 1991). Freeman (1995) used *in situ* hybridization to detect the mRNA abundance of *SGLT1* in rabbit intestine and found that it was highest in the ileum, followed by the jejunum and duodenum. Our study showed that the mRNA levels of *SGLT1* in the ileum of Jinhua pigs were significantly lower. This may suggest that compared with Landrace pigs, the transcription levels of the glucose transporter genes in Jinhua pigs maybe lower.

Fatty acid-binding proteins are found to be closely related to fat absorption, transportation, and metabolism (Besnard et al., 2002; Tan et al., 2014; Byrne et al., 2015). *FABP1* and *FABP2* are essential in the β-oxidation of unesterified fatty acids and long-chain fatty acids, respectively while *FABP4* could regulate intracellular lipid transport in various tissue (Spiegelman et al., 1983; Hittel and Storey, 2002; Liu et al., 2008; Montoudis et al., 2008). *FABP2* has a high affinity for long-chain fatty acids (Ho and Storch, 2001). The body weight of mice with a *FABP2* deletion was significantly lower than normal mice fed high-fat diets (Vassileva et al., 2000). Our study showed that the intestinal lipase activity of Jinhua pigs was higher than that of Landrace pigs, while the *FABP1*, *FABP2*, and *FABP4* in all segments of the small intestinal mucosa of Jinhua pigs were expressed at higher levels than those in Landrace pigs. These data indicate that Jinhua pigs may be superior to Landrace pigs in the digestion and absorption of fatty acids.

## Conclusion

Collectively, the activities of digestive enzymes and the expression levels of nutrient transporters were tissue-specific and species-specific. The difference in the activities of digestive enzymes and the expression levels of nutrient transporters between Jinhua and Landrace pigs might partially explain why the growth performance and fat deposition of Jinhua and Landrace pigs are different.

## Data Availability Statement

The raw data supporting the conclusions of this article will be made available by the authors, without undue reservation.

## Ethics Statement

The animal study was reviewed and approved by Institutional Animal Care and Use Committee of the Zhejiang Academy of Agricultural Sciences.

## Author Contributions

XL, WL, LL, and YX: conception and design of study. XL, WL, LL, KL, FZ, YW, JC, BD, HY, and YX: acquisition of data. XL, WL, LL, KL, and FZ: analysis and/or interpretation of data. XL: drafting the manuscript. BD, HY, and YX: revising the manuscript critically for important intellectual content. All authors contributed to the article and approved the submitted version.

## Conflict of Interest

LL and KL were employed by the company Zhejiang Goshine Test Technologies Co., Ltd. BD was the boss of the company Zhejiang Goshine Test Technologies Co., Ltd. The remaining authors declare that the research was conducted in the absence of any commercial or financial relationships that could be construed as a potential conflict of interest.

## Abbreviations

*FABP1*: fatty acid-binding protein 1
*FABP2*: fatty acid-binding protein 2
*FABP4*: fatty acid-binding protein 4
*FABPs*: fatty acid-binding proteins
*GLUT2*: glucose transporter 2
*PepT1*: oligopeptide transporter 1
*SGLT1*: sodium glucose cotransporter.

## References

[B1] Adeola O. King D. E. (2006). Developmental changes in morphometry of the small intestine and jejunal sucrase activity during the first nine weeks of postnatal growth in pigs. *J. Anim. Sci*. 84 112–118. 10.2527/2006.841112x 16361497

[B2] Adibi S. A. (2003). Regulation of expression of the intestinal oligopeptide transporter (Pept-1) in health and disease. *Am. J. Physiol. Gastrointest. Liver Physiol*. 285 779–788. 10.1152/ajpgi.00056.2003 14561585

[B3] Baker D. H. (2008). Animal models in nutrition research. *J. Nutr.* 138 391–396. 10.1093/jn/138.2.391 18203909

[B4] Barea R. Nieto R. Vitari F. Domeneghini C. Aguilera J. F. (2011). Effects of pig genotype (Iberian v. Landrace x Large White) on nutrient digestibility, relative organ weight and small intestine structure at two stages of growth. *Animal* 5 547–557. 10.1017/S1751731110002181 22439951

[B5] Beck I. T. (1973). The role of pancreatic enzymes in digestion. *Am. J. Clin. Nutr*. 26 311–325. 10.1093/ajcn/26.3.311 4347665

[B6] Besnard P. Niot I. Poirier H. Clement L. Bernard A. (2002). New insights into the fatty acid-binding protein (*FABP*) family in the small intestine. *Mol. Cell Biochem*. 239 139–147. 10.1007/978-1-4419-9270-3_18 12479579

[B7] Byrne C. S. Chambers E. S. Morrison D. J. Frost G. (2015). The role of short chain fatty acids in appetite regulation and energy homeostasis. *Int. J. Obes.* 39 1331–1338. 10.1038/ijo.2015.84 25971927 PMC4564526

[B8] Cera K. R. Mahan D. C. Reinhart G. A. (1990). Effect of weaning, week postweaning and diet composition on pancreatic and small intestinal luminal lipase response in young swine. *J. Anim. Sci*. 68 384–391. 10.2527/1990.682384x 2312429

[B9] Courtois P. Meuris S. Sener A. Malaisse W. J. Scott F. W. (2002). Invertase, maltase, lactase, and peroxidase activities in duodenum of BB rats. *Endocrine* 19 293–300. 10.1385/ENDO:19:3:293 12624429

[B10] Deng B. Wu J. Li X. Zhang C. Men X. Xu Z. (2020). Effects of Bacillus subtilis on growth performance, serum parameters, digestive enzyme, intestinal morphology, and colonic microbiota in piglets. *AMB Exp.* 10 1–10. 10.1186/S13568-020-01150-Z 33263814 PMC7710768

[B11] Fei Y. J. Kanai Y. Nussberger S. Ganapathy V. Leibach F. H. Romero M. F. (1994). Expression cloning of a mammalian proton-coupled oligopeptide transporter. *Nature* 368 563–566. 10.1038/368563a0 8139693

[B12] Freeman T. C. (1995). Parallel patterns of cell-specific gene expression during enterocyte differentiation and maturation in the small intestine of the rabbit. *Differentiation* 59 179–192. 10.1046/j.1432-0436.1995.5930179.x 7589902

[B13] Gan M. Shen L. Chen L. Jiang D. Jiang Y. Li Q. (2020). Meat quality, amino acid, and fatty acid composition of Liangshan pigs at different weights. *Animals* 10:822. 10.3390/ani10050822 32397391 PMC7278381

[B14] Hittel D. S. Storey K. B. (2002). The translation state of differentially expressed mRNAs in the hibernating 13-lined ground squirrel (*Spermophilus tridecemlineatus*). *Arch. Biochem. Biophys*. 401 244–254. 10.1016/S0003-9861(02)00048-6 12054475

[B15] Ho S. Y. Storch J. (2001). Common mechanisms of monoacylglycerol and fatty acid uptake by human intestinal Caco-2 cells. *Am. J. Physiol. Cell Physiol*. 281 1106–1117. 10.1152/ajpcell.2001.281.4.C1106 11546646

[B16] Huang M. Chen L. Shen Y. Chen J. Guo X. Xu N. (2019). Integrated mRNA and miRNA profile expression in livers of Jinhua and Landrace pigs. *Asian-Australas J. Anim. Sci*. 32 1483–1490. 10.5713/ajas.18.0807 31010989 PMC6718901

[B17] Jensen M. S. Jensen S. K. Jakobsen K. (1997). Development of digestive enzymes in pigs with emphasis on lipolytic activity in the stomach and pancreas. *J. Anim. Sci*. 75 437–445. 10.2527/1997.752437x 9051467

[B18] Kemp B. L. Hartog L. A. Klok J. J. Zandstra T. (1991). The digestibility of nutrients, energy and nitrogen in the Meishan and Dutch Landrace pig. *J. Anim. Physiol. Anim. Nutr.* 65 263–266. 10.1111/j.1439-0396.1991.tb00265.x

[B19] King D. E. Asem E. K. Adeola O. (2000). Ontogenetic development of intestinal digestive functions in White Pekin ducks. *J. Nutr*. 130 57–62. 10.1093/jn/130.1.57 10613767

[B20] Kluge H. Broz J. Eder K. (2006). Effect of benzoic acid on growth performance, nutrient digestibility, nitrogen balance, gastrointestinal microflora and parameters of microbial metabolism in piglets. *J. Anim. Physiol. Anim. Nutr.* 90 316–324. 10.1111/j.1439-0396.2005.00604.x 16867077

[B21] Liang R. Fei Y. J. Prasad P. D. Ramamoorthy S. Han H. Yang-Feng T. L. (1995). Human intestinal H+/peptide cotransporter. Cloning, functional expression, and chromosomal localization. *J. Biol. Chem*. 270 6456–6463. 10.1074/jbc.270.12.6456 7896779

[B22] Lindemann M. D. Cornelius S. G. el Kandelgy S. M. Moser R. L. Pettigrew J. E. (1986). Effect of age, weaning and diet on digestive enzyme levels in the piglet. *J. Anim. Sci*. 62 1298–1307. 10.2527/jas1986.6251298x 2424884

[B23] Liu R. Z. Li X. Godbout R. (2008). A novel fatty acid-binding protein (FABP) gene resulting from tandem gene duplication in mammals: transcription in rat retina and testis. *Genomics*. 92 436–445. 10.1016/j.ygeno.2008.08.003 18786628

[B24] Livak K. J. Schmittgen T. D. (2001). Analysis of relative gene expression data using real-time quantitative PCR and the 2(-Delta Delta C(T)) Method. *Methods* 25 402–408. 10.1006/meth.2001.1262 11846609

[B25] Makkink C. A. Berntsen P. J. op den Kamp B. M. Kemp B. Verstegen M. W. (1994). Gastric protein breakdown and pancreatic enzyme activities in response to two different dietary protein sources in newly weaned pigs. *J. Anim. Sci*. 72 2843–2850. 10.2527/1994.72112843x 7730177

[B26] Martins J. M. Fialho R. Albuquerque A. Neves J. Freitas A. Nunes J. T. (2020). Growth, blood, carcass and meat quality traits from local pig breeds and their crosses. *Animal* 14 636–647. 10.1017/S1751731119002222 31578161

[B27] Miao Z. G. Wang L. J. Xu Z. R. Huang J. F. Wang Y. R. (2009). Developmental changes of carcass composition, meat quality and organs in the Jinhua pig and Landrace. *Animal* 3 468–473. 10.1017/S1751731108003613 22444318

[B28] Miller E. R. Ullrey D. E. (1987). The Pig as a model for human nutrition. *Annu. Rev. Nutr*. 7 361–382. 10.1146/annurev.nu.07.070187.002045 3300739

[B29] Montoudis A. Seidman E. Boudreau F. Beaulieu J. F. Menard D. Elchebly M. (2008). Intestinal fatty acid binding protein regulates mitochondrion beta-oxidation and cholesterol uptake. *J. Lipid Res*. 49 961–972. 10.1194/jlr.M700363-JLR200 18235139

[B30] Nichols B. L. Avery S. Sen P. Swallow D. M. Hahn D. Sterchi E. (2003). The maltase-glucoamylase gene: common ancestry to sucrase-isomaltase with complementary starch digestion activities. *Proc. Natl. Acad. Sci. U S A*. 100 1432–1437. 10.1073/pnas.0237170100 12547908 PMC298790

[B31] Owsley W. F. (1982). Effects of age and diet on digestive function in the young pig. *Texas Tech University* 66 676–693. 10.1210/endo-66-5-676 13852038

[B32] Riby J. E. Kretchmer N. (1985). Participation of pancreatic enzymes in the degradation of intestinal sucrase-isomaltase. *J. Pediatr. Gastroenterol. Nutr*. 4 971–979. 10.1097/00005176-198512000-00020 3906077

[B33] Samulitis-dos Santos B. K. Goda T. Koldovsky O. (1992). Dietary-induced increases of disaccharidase activities in rat jejunum. *Br. J. Nutr*. 67 267–278. 10.1079/bjn19920030 1596499

[B34] Semenza G. (1986). Anchoring and biosynthesis of stalked brush border membrane proteins: glycosidases and peptidases of enterocytes and renal tubuli. *Annu. Rev. Cell Biol*. 2 255–313. 10.1146/annurev.cb.02.110186.001351 3548768

[B35] Spiegelman B. M. Frank M. Green H. (1983). Molecular cloning of mRNA from 3T3 adipocytes. Regulation of mRNA content for glycerophosphate dehydrogenase and other differentiation-dependent proteins during adipocyte development. *J. Biol. Chem*. 258 10083–10089. 10.1016/S0021-9258(17)44608-4 6411703

[B36] Smoot J. C. Findlay R. H. (2000). Digestive enzyme and gut surfactant activity of detritivorous gizzard shad (Dorosoma cepedianum). *Can. J. Fish. Aquat. Sci*. 57 1113–1119. 10.1139/f00-036 33356898

[B37] Swallow D. M. Poulter M. Hollox E. J. (2001). Intolerance to lactose and other dietary sugars. *Drug. Metab. Dispos*. 29 513–516. 10.1016/S1359-6446(01)01691-9 11259342

[B38] Shinohara H. Tsuji Y. Yamada K. Hosoya N. (1986). Effects of carbohydrate intake on disaccharidase activity and disaccharide-evoked transmural potential difference in rat small intestine. *Nippon. Eiyo. Shokuryo. Gakkaishi.* 39 35–41. 10.4327/jsnfs.39.35

[B39] Tan J. McKenzie C. Potamitis M. Thorburn A. N. Mackay C. R. Macia L. (2014). The role of short-chain fatty acids in health and disease. *Adv. Immunol*. 121 91–119. 10.1016/B978-0-12-800100-4.00003-9 24388214

[B40] Tavakkolizadeh A. Ramsanahie A. Levitsky L. L. Zinner M. J. Whang E. E. Ashley S. W. (2005). Differential role of vagus nerve in maintaining diurnal gene expression rhythms in the proximal small intestine. *J. Surg. Res*. 129 73–78. 10.1016/j.jss.2005.05.023 16087191

[B41] Uni Z. Ganot S. Sklan D. (1998). Posthatch development of mucosal function in the broiler small intestine. *Poult. Sci*. 77 75–82. 10.1093/ps/77.1.75 9469755

[B42] Urriola P. E. Stein H. H. (2012). Comparative digestibility of energy and nutrients in fibrous feed ingredients fed to Meishan and Yorkshire pigs. *J. Anim. Sci*. 90 802–812. 10.2527/jas.2010-3254 21984712

[B43] Vassileva G. Huwyler L. Poirier K. Agellon L. B. Toth M. J. (2000). The intestinal fatty acid binding protein is not essential for dietary fat absorption in mice. *FASEB J*. 14 2040–2046. 10.1096/fj.99-0959com 11023988

[B44] Ville E. Carriere F. Renou C. Laugier R. (2002). Physiological study of pH stability and sensitivity to pepsin of human gastric lipase. *Digestion* 65 73–81. 10.1159/000057708 12021480

[B45] Vincenzini M. T. Iantomasi T. Favilli F. (1989). Glutathione transport across intestinal brush-border membranes: effects of ions, pH, delta psi, and inhibitors. *Biochim. Biophys. Acta*. 987 29–37. 10.1016/0005-2736(89)90451-3 2597684

[B46] Wang Y. Thakali K. Morse P. Shelby S. Chen J. Apple J. (2021). Comparison of growth performance and meat quality traits of commercial cross-bred pigs versus the large black pig breed. *Animals* 11:200. 10.3390/ani11010200 33467586 PMC7830199

[B47] Wright E. M. Turk E. Zabel B. Mundlos S. Dyer J. (1991). Molecular genetics of intestinal glucose transport. *J. Clin. Invest*. 88 1435–1440. 10.1172/JCI115451 1939637 PMC295642

[B48] Wu T. Zhang Z. Yuan Z. Lo L. J. Chen J. Wang Y. (2013). Distinctive genes determine different intramuscular fat and muscle fiber ratios of the longissimus dorsi muscles in Jinhua and landrace pigs. *PLoS One* 8:e53181. 10.1371/journal.pone.0053181 23301040 PMC3536781

[B49] Wu C. Lyu W. Hong Q. Zhang X. Yang H. Xiao Y. (2021). Gut microbiota influence lipid metabolism of skeletal muscle in pigs. *Front. Nutr.* 8:675445. 10.3389/fnut.2021.675445 33928112 PMC8076524

[B50] Xiao Y. Kong F. Xiang Y. Zhou W. Wang J. Yang H. (2018). Comparative biogeography of the gut microbiome between Jinhua and Landrace pigs. *Sci. Rep*. 8:5985. 10.1038/s41598-018-24289-z 29654314 PMC5899086

[B51] Yen J. T. Nienaber J. A. Hill D. A. Pond W. G. (1991). Potential contribution of absorbed volatile fatty acids to whole-animal energy requirement in conscious swine. *J. Anim. Sci.* 69 2001–2012. 10.2527/1991.6952001 2066310

