# Supplementary material for: Comparison of Digestive Enzyme Activities and Expression of Small Intestinal Transporter Genes in Jinhua and Landrace Pigs
Source: Front Physiol. 2021 Jun 14;12:669238. doi: 10.3389/fphys.2021.669238 (PMC8236719; doi:10.3389/fphys.2021.669238)
Supplement: Supplementary file 1 [file Table_1.docx]

**Table 1** Primer information for genes chosen for confirmation of expression using RT-PCR

| Gene Name | Gene Library Sequence Number | Primer sequence (5' to 3')  (F:former primer,R:reverse primer) | Size(bp) |
| --- | --- | --- | --- |
|  |  |  |  |
| Pept1 | NM_214347.1 | F:GCAGACCGTCAACGCCATCCT | 125 |
|  |  | R:GGAACATCCCAACTGTCATCTTCCT |  |
| SGLT1 | NM_001164021.1 | F:CCCAGCAACTGTCCCACAATT | 135 |
|  |  | R:GCGGTAGAGATGCACATCTGGAA |  |
| GLUT2 | NM_001097417.1 | F:CGGTGGGACTTGTGCTACTGGA | 146 |
|  |  | R:GCGTGGTCCTTGACTGAAAAACT |  |
| FABP1 | NM_001004046.2 | F:TGAACTCAACGGTGACATA | 75 |
|  |  | R:ATTCTCTTGCTGATTCTCTTG |  |
| FABP2 | NM_001031780.1 | F:CTCGCAGACGGAACTGAACTCA | 127 |
|  |  | R:CCATTTCATCCCCGATAATTTCT |  |
| FABP4 | NM_001002817.1 | F:TGGAAACTTGTCTCCAGTG | 147 |
|  |  | R:GGTACTTTCTGATCTAATGGTG |  |
| GAPDH | AF017079 | F:GGCAAATTCCACGGCACAGTCA | 82 |
|  |  | R:CTCGCTCCTGGAAGATGGTGAT |  |
|  |  |  |  |
